# Choroidal fissure acts as an overflow device in cerebrospinal fluid drainage: morphological comparison between idiopathic and secondary normal-pressure hydrocephalus

**DOI:** 10.1038/srep39070

**Published:** 2016-12-12

**Authors:** Shigeki Yamada, Masatsune Ishikawa, Yasushi Iwamuro, Kazuo Yamamoto

**Affiliations:** 1 Normal Pressure Hydrocephalus Center, Rakuwakai Otowa Hospital, Kyoto, Japan; 2 Department of Neurosurgery and Stroke Center, Rakuwakai Otowa Hospital, Kyoto, Japan

## Abstract

To clarify the pathogenesis of two different types of adult-onset normal-pressure hydrocephalus (NPH), we investigated cerebrospinal fluid distribution on the high-field three-dimensional MRI. The subarachnoid spaces in secondary NPH were smaller than those in the controls, whereas those in idiopathic NPH were of similar size to the controls. In idiopathic NPH, however, the basal cistern and Sylvian fissure were enlarged in concurrence with ventricular enlargement towards the z-direction, but the convexity subarachnoid space was severely diminished. In this article, we provide evidence that the key cause of the disproportionate cerebrospinal fluid distribution in idiopathic NPH is the compensatory direct CSF communication between the inferior horn of the lateral ventricles and the ambient cistern at the choroidal fissure. In contrast, all parts of the subarachnoid spaces were equally and severely decreased in secondary NPH. Blockage of CSF drainage from the subarachnoid spaces could cause the omnidirectional ventricular enlargement in secondary NPH.

Since 1965, adult-onset normal pressure hydrocephalus (NPH) has been largely classified into idiopathic NPH (iNPH) or secondary NPH (sNPH), which develops after subarachnoid haemorrhage, trauma or infection^1^. The diagnosis and management of iNPH has made rapid progress since evidence-based guidelines were established^2^,^3^,^4^,^5^,^6^. The most typical morphological characteristics of CSF distribution in iNPH were narrow sulci at the high convexity due to z-axial expansion of the lateral ventricles and enlargement of the basal cistern and Sylvian fissure^7^,^8^,^9^,^10^,^11^. Subsequently, many MRI volumetric analyses proposed several parameters specific to iNPH^9^,^10^,^12^,^13^,^14^. Nevertheless, little information is available on the CSF distribution in sNPH. Therefore, we quantitatively assessed the CSF distributions in patients with sNPH compared with those in patients with iNPH by using volumetric analysis based on the high-field T2-weighted 3D MRI. Recently, we provided new evidence for the comparison of the CSF volumes observed in patients with iNPH and those with Alzheimer disease^10^. Here, we reported that the basal cistern and Sylvian fissure in patients with iNPH was enlarged having the same mean volume as that in patients with Alzheimer disease, and, in addition the ventricles in iNPH patients were >40 mL larger than those in Alzheimer disease. These findings contribute to our understanding of the development of concurrent expansion of the ventricles and basal cistern and Sylvian fissure in iNPH. Compensatory direct CSF pathways between ventricles and subarachnoid spaces other than the foramina of Luschka and Magendie have been observed in hydrocephalic rat models^15^,^16^,^17^,^18^. Therefore, we hypothesized that the choroidal fissure, which is formed from the medial wall of the lateral ventricles, was opened and directly mediated the flow of CSF between the lateral ventricles and the basal cistern, acting as an overflow device under conditions of increased intracranial CSF volume, such as in iNPH. To elucidate the pathogenesis of iNPH and sNPH, we investigated this direct communication between the ventricles and the basal cistern, focusing on the inferior choroidal point of the choroidal fissure.

## Results

### Clinical characteristics and morphological indices

Fifty-two patients were diagnosed with iNPH, 15 patients were diagnosed with sNPH, and 31 participants were allocated to the control group. The mean ages were not significantly different among the three groups (Table 1). The mean time of disease duration from the initial presentation of symptoms until the diagnosis of iNPH was 2 years. The mean duration from the initial event of subarachnoid haemorrhage or head injury until the diagnosis of sNPH was 5 months. Evans index >0.3, which is the most popular index of ventricular enlargement^2^,^3^,^4^,^5^,^6^, was satisfied by approximately 80% of the patients diagnosed both with iNPH and sNPH. In contrast, z-Evans index >0.4^9^, callosal angle <90°^19^, high convexity tightness and enlarged Sylvian fissure^7^,^8^,^11^, which have been reported to be specific indices of iNPH, were satisfied in ≥85% of patients with iNPH but in <55% of those with sNPH (Table 1); this difference between the two groups was statistically significant. Severe periventricular hyperintensity (smooth halo or expanding) and severe deep white matter hyperintensity (confluence of foci) on the Fazekas rating scale^20^ were frequently observed in the patients with iNPH, whereas the patients with sNPH had similar percentages of hyperintensity when compared to the controls (Table 1). Additionally, an opening of the inferior choroidal point, which we here newly define as a direct pathway linking CSF spaces between the inferior horn of the lateral ventricles and the ambient cistern at the inferior choroidal point of the choroidal fissure, was observed in 39 of 52 patients (75%) with iNPH but in only 2 of 11 (13%) with sNPH and in none of the controls (Table 1).

### Comparison of ventricular shape and CSF volumes between iNPH and sNPH

**Figure 1:**
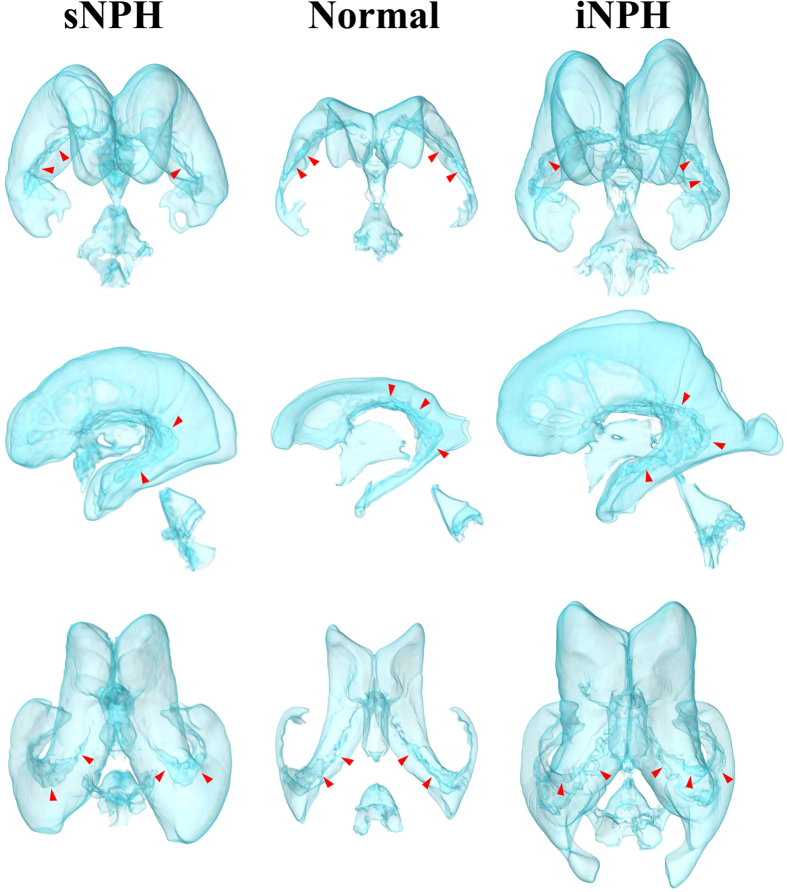
Three-dimensional views of the ventricles in iNPH, sNPH and normal brain. Compared to the controls (middle), the ventricles in sNPH patients expanded symmetrically in all directions, whereas the ventricles in iNPH patients expanded disproportionately towards the z-axial direction. The red arrow heads indicate the choroid plexus.

**Figure 2:**
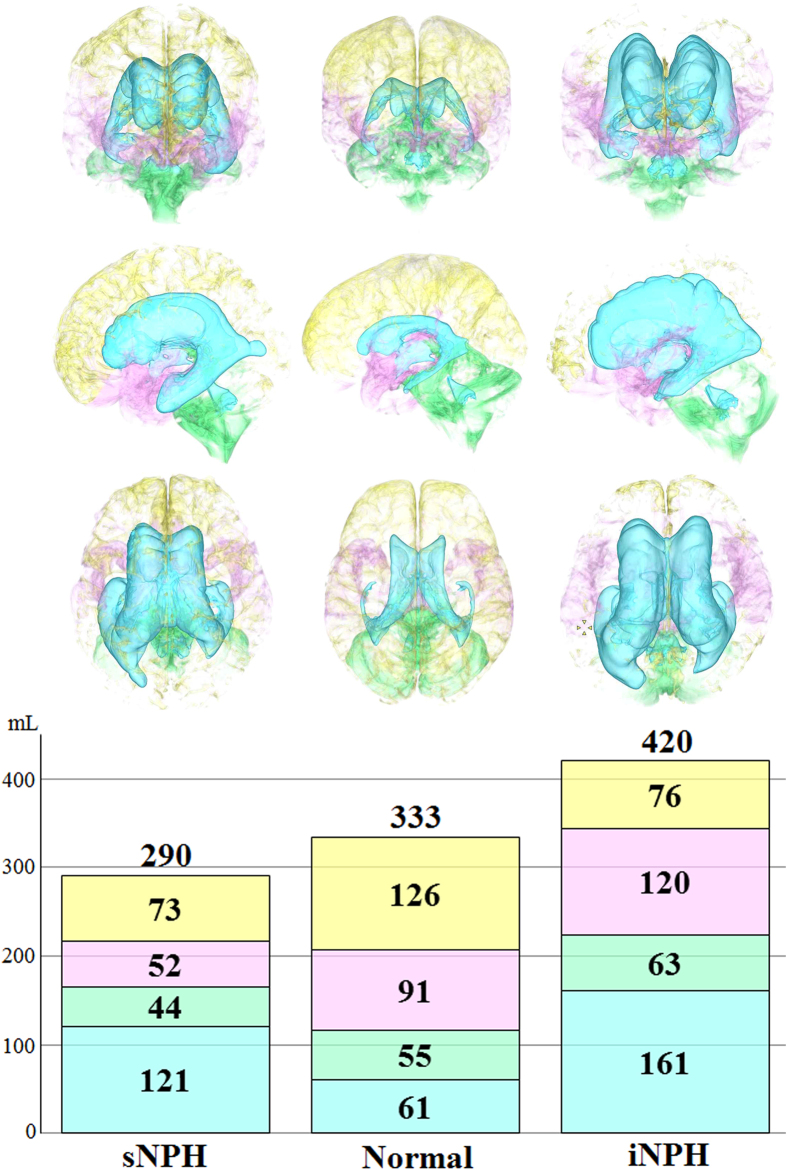
Mean volumes of the segmented intracranial cerebrospinal fluid spaces in iNPH, sNPH and normal brain. The mean volumes of ventricles (sky blue), basal cistern and Sylvian fissure (pink) and subarachnoid space in the posterior fossa (light green) were the largest in patients with iNPH. The mean volume of the convexity part of the subarachnoid space (light yellow) was equally decreased in patients with iNPH and those with sNPH.

Figure 1 shows the ventricles in the representative cases with iNPH and sNPH and in the control groups. The total size of all the ventricles in patients with iNPH and sNPH were 2.6 and 2.0 times larger, respectively, than those in the controls (Table 2). The mean volume and volume ratio of the total subarachnoid spaces in patients with iNPH and controls were almost identical (260 mL and 17% verses 272 mL and 18%, respectively), however, the CSF distribution in the subarachnoid spaces was different between these two groups, as shown in Fig. 2 and Table 2. The mean volume of the basal cistern and Sylvian fissure in patients with iNPH was 30 mL larger than that in the controls, whereas the volume of the convexity subarachnoid space was 50 mL smaller in patients with iNPH than in the controls. Furthermore, we observed that each of the three parts of the subarachnoid spaces in patients with sNPH was significantly smaller than each of those parts in the controls by equal proportions, and the mean volume of the total subarachnoid spaces in patients with sNPH was >100 mL smaller than that in the controls. Overall, the intracranial CSF spaces in patients with iNPH were significantly larger than those in the age-matched controls, whereas those in patients with sNPH were smaller (Fig. 2 and Table 2).

### Patients with iNPH possess a direct CSF pathway at the inferior choroidal point of the choroidal fissure

**Figure 3:**
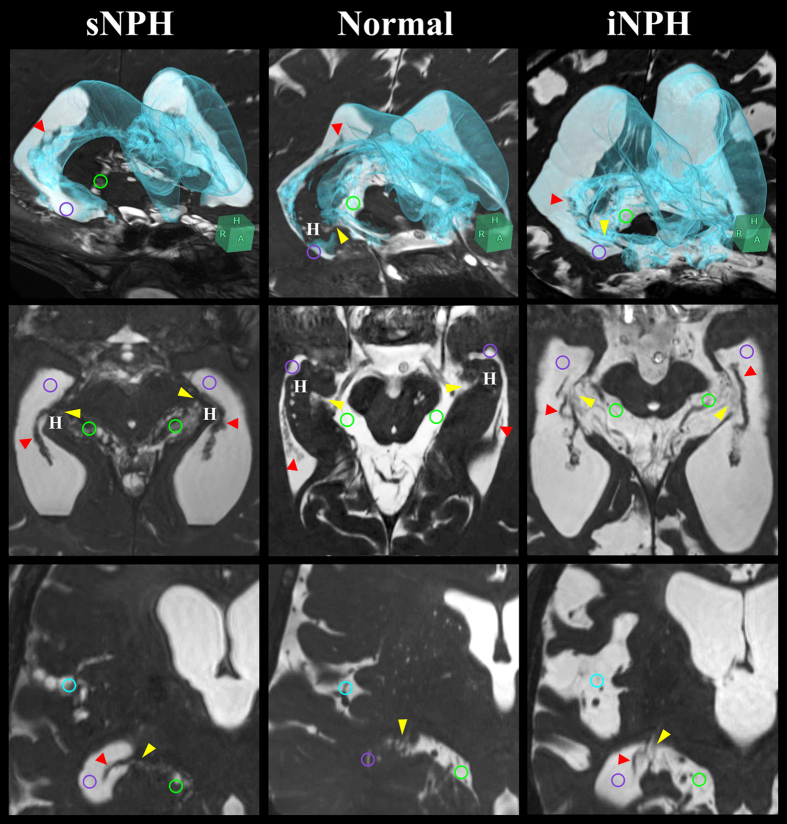
Fine structures of the lateral ventricles and the basal cistern around the inferior choroidal point of the choroidal fissure. The 3D views (upper) which combine axial (middle) and coronal (lower) sections were at the level of the inferior choroidal point of the choroidal fissure (yellow arrow head) between the ambient cistern (light green circle) and the inferior horn of the lateral ventricles (purple circle). In the normal situation, at the head of the hippocampus (H) and choroid plexus (red arrow head), the ambient cistern is separated from the inferior horn of the lateral ventricles. In patients with iNPH, the ventricles, basal cistern and Sylvian fissure (light blue circle) were enlarged with the concurrent opening of the inferior choroidal point of the choroidal fissure. However, in patients with sNPH, only the ventricle was enlarged and all of the subarachnoid spaces, including the ambient cistern and Sylvian fissure, were severely diminished.

Figure 3 shows key sections on 3D-constructive interference in steady state (CISS) MRI sequences from representative patients with iNPH or sNPH and control groups. Compared to the controls, all regions of subarachnoid space, including the ambient cistern and Sylvian fissure, were severely diminished in the patients with sNPH, although the ventricles were enormously enlarged. On the contrary, not only the ventricles but also the Sylvian fissure and basal cistern, including the ambient cistern, were conspicuously enlarged in the patients with iNPH. Normally, the choroidal fissure at the medial wall of the lateral ventricles is constructed from the choroid plexus and a thin membrane without ependyma^21^,^22^. At the inferior choroidal point of the choroidal fissure, the anterior choroid arteries and inferior choroidal veins run through the ambient cistern and the inferior horn of the lateral ventricles. In the patients with iNPH, however, the choroid plexus appeared to float in the CSF, and the border seemed to disappear due to the enlargement of the ambient cistern and inferior horn and the upward displacement and compression of the head of the hippocampus (Fig. 3, right).

**Figure 4:**
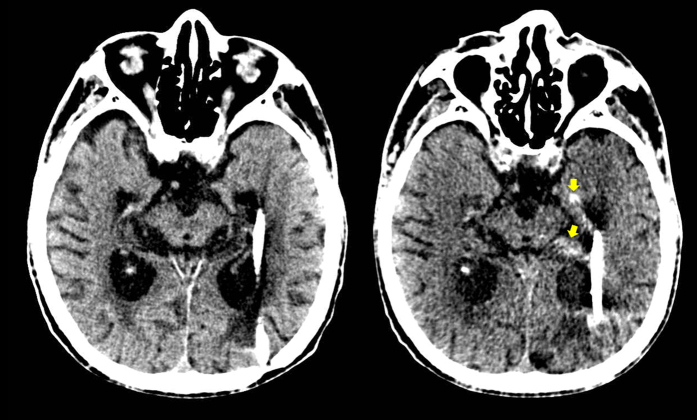
Confirmation of the direct CSF communication between the lateral ventricles and the ambient cistern by shuntography in patients with iNPH with a suspected shunt malfunction. Contrast medium (yellow arrow head) was observed not only in the left inferior horn of the lateral ventricles but also in the left ambient cistern on CT scan immediately after shuntography (right), compared to that before shuntography (left).

Further evidence indicating a direct pathway of CSF flow at the inferior choroidal point of the choroidal fissure is presented in head CT images taken before and immediately after shuntography performed on a patient with iNPH with a suspected shunt malfunction (Fig. 4). On the CT image taken immediately after shuntography in the lower right lateral decubitus position, contrast medium was observed in both the inferior horn of the lateral ventricles and in the ambient cistern.

In the video clip showing an amygdalohippocampectomy (Supplementary), it is evident that, before microsurgical dissection of the transparent membrane of the choroidal fissure, the CSF in the ambient cistern passed through this membrane and moved freely in and out of the inferior horn of the lateral ventricles. This to-and-fro movement of the CSF provides evidence that the fluid could freely penetrate the thin arachnoid membrane at the inferior choroidal point of the choroidal fissure. However, these additional clinical findings are not sufficient to prove direct CSF communication at the inferior choroidal point of the choroidal fissure because each finding was verified in only one patient.

## Discussion

**Figure 5:**
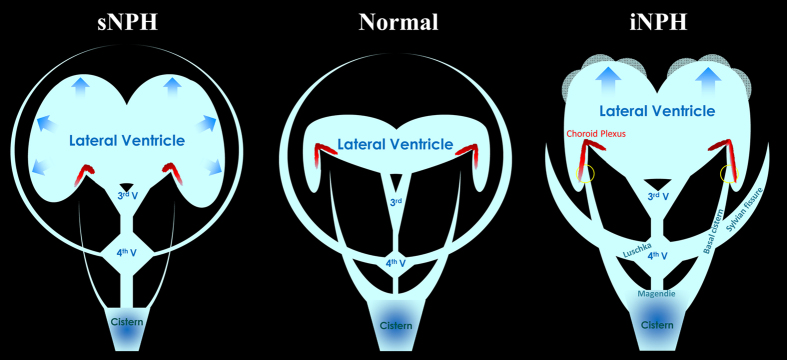
Schematic diagram showing the distribution of CSF in patients with iNPH and sNPH, compared to normal brains. In iNPH patients, the inferior horn of the lateral ventricles is directly connected with the basal cistern at the inferior choroidal point of the choroidal fissure (yellow circle) inside the choroid plexus.

This study shows that CSF distribution markedly differs between the two categories of NPH. Figure 5 shows schemata representing intracranial CSF distributions in iNPH-affected, sNPH-affected and normal brain. The most distinct morphological characteristics in the patients with iNPH were z-axial expansion of the bilateral ventricles in concurrence with enlargement of the basal cistern and Sylvian fissure and diminishment of the convexity subarachnoid spaces. In contrast, the patients with sNPH exhibited ventricles that were enlarged symmetrically in all directions in concurrence with each of the three subarachnoid spaces being severely diminished. The morphological discrepancies between iNPH and sNPH lead us to conclude that the pathogenesis of iNPH is markedly different from the developmental process of sNPH following subarachnoid haemorrhage or brain injury.

Recently, classical understanding of CSF bulk flow and the concept of the third circulation have changed^23^,^24^,^25^,^26^,^27^,^28^,^29^,^30^,^31^. In the concept of third circulation^32^,^33^,^34^, CSF mainly secreted by the choroid plexus in the ventricles moves as unidirectional bulk flow from the ventricles to subarachnoid spaces through the foramina of Luschka and Magendie and finally absorbs from the arachnoid villi at the superior sagittal sinus. In contrast, recent experimental data have provided evidence that the choroid plexus is not the major site of CSF production and parenchymal interstitial fluid is excreted into the ventricles and subarachnoid spaces as CSF^25^,^26^,^27^,^29^,^30^,^31^,^35^,^36^. Ichimura *et al*. reported that the direction of the interstitial fluid flow towards the ventricles or subarachnoid spaces varies in an unpredictable manner^35^. Furthermore, current research suggests that lymphatic drainage via the peri- and para-vascular space^23^,^31^,^37^,^38^,^39^,^40^, perineural subarachnoid spaces of the cranial nerves or meninges^25^,^26^,^41^,^42^ is the major pathway for absorption of CSF and interstitial fluid, and venous drainage via the dural arachnoid villi is the minor^23^,^24^,^28^,^43^. Newly developed MRI techniques for assessing CSF dynamics have revealed that CSF moves in a pulsatile fashion and not in a unidirectional bulk flow^44^,^45^,^46^,^47^,^48^,^49^,^50^. CSF moves upward and downward around the cranio-cervical junction with cardiac pulsation and respiration as driving forces^44^,^45^,^46^,^47^,^48^,^49^,^50^. These recent findings have negated the classical concept of the CSF bulk flow from the ventricles to subarachnoid spaces through the foramina of Luschka and Magendie.

**Figure 6:**
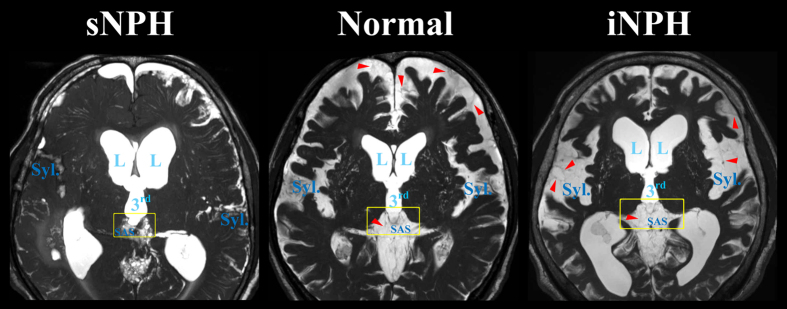
Fine structures of the subarachnoid spaces around the third ventricle. Patients with iNPH had the enlarged Sylvian fissure (Syl.) in concurrent with enlargement of the lateral (L) and third (3^rd^) ventricles, whereas patients with sNPH had the diminished Sylvian fissure. The border between third ventricle and surrounding subarachnoid spaces (SAS) was thinning in iNPH patients (yellow square). The red arrow heads indicate the trabeculas and partitions in the subarachnoid spaces.

Our observation of CSF distribution reduced subarachnoid spaces in the high parietal convexity in iNPH supports the current view of CSF absorption. The main pathogenic mechanism associated with iNPH is thought to be the disruption of the most downstream site of CSF drainage systems, because patients with iNPH have large volumes of intracranial CSF. The subarachnoid spaces might be harder to expand than the ventricles because there are many trabeculas and partitions in the subarachnoid spaces, as shown in Fig. 6. Additionally, the most downstream site for CSF absorption is likely to become the most enlarged CSF spaces which were the ventricles, basal cistern and Sylvian fissure, but not convexity subarachnoid space in iNPH. We have also produced new evidence that the ventricles and subarachnoid space possess several direct alternative connections other than the foramina of Luschka and Magendie that allow protection from overflow^15^,^16^,^17^,^18^. The expansion of intracranial CSF volume in iNPH creates compensatory routes between the ventricles and subarachnoid spaces. Mainly, the inferior horn of the lateral ventricles directly communicates with the ambient cistern via the opening of the inferior choroidal point of the choroidal fissure, serving as an overflow management point for the ventricular drainage system. However, the choroidal fissure appears open in most, but not all, patients with iNPH. In a hydrocephalic rat model, Park *et al*. found that the interface between the third ventricle and the quadrigeminal cistern also serves as a compensatory direct CSF pathway in addition to the choroidal fissure^17^. We confirmed that the subarachnoid spaces around the third ventricle appear enlarged in patients with iNPH and diminished in patients with sNPH compared to a control population (Fig. 6). Further investigations are needed to confirm the other compensatory direct CSF pathways in iNPH patients with a closed choroidal fissure.

On the other hand, the main pathogenic factor for the omnidirectional expansion of ventricles in sNPH is thought to be the disruption of CSF drainage from subarachnoid spaces, because the subarachnoid spaces in sNPH extensively diminished and the total intracranial CSF volume was smaller than that in the age-matched controls. Some clinical studies have reported that significantly increased inflammatory fibrogenic cytokines or ferritin levels in CSF are associated with sNPH after subarachnoid haemorrhage^51^,^52^,^53^. Additionally, a rat model of communicating hydrocephalus by injecting kaolin in the subarachnoid spaces mimics human sNPH^54^,^55^,^56^. In a kaolin-induced sNPH model, the subarachnoid spaces were obstructed with the number of macrophages and severe fibrosis^55^,^56^. Inflammation in CSF is known to be associated with arachnoid and pia matter fibrosis^57^, which might lead to the closure of subarachnoid spaces. The present findings serve as a foundation for further investigation into the pathophysiological mechanisms underlying the development of iNPH and sNPH.

In conclusion, the present study revealed the characteristics of CSF distribution in patients with iNPH, which strongly differed from those in patients with sNPH. Patients with iNPH had a substantially larger total CSF volume than control subjects, whereas patients with sNPH had a smaller total CSF volume. Under normal conditions, the ventricles directly communicate with the subarachnoid spaces via the foramina of Luschka and Magendie. However, in iNPH, the ventricles and basal cistern are enlarged, along with the opening of the inferior choroidal point of the choroidal fissure. This provides a direct compensatory CSF communication route between the ventricles and subarachnoid spaces. Thus, this is a key cause of the disproportionate CSF distribution in iNPH, named “Disproportionately Enlarged Subarachnoid-space Hydrocephalus (DESH)”^7^, which is z-axial expansion of the bilateral ventricles in concurrence with enlargement of the basal cistern and Sylvian fissure and diminishment of the convexity subarachnoid spaces. Conversely, in sNPH all parts of the subarachnoid spaces are equally and severely reduced in size. We postulate that fibrosis of arachnoid and pia matter reduces the subarachnoid spaces leading to expansion of the ventricles. These novel findings may contribute to future studies of the mechanisms that drive CSF movement and absorption.

## Methods

### Ethical approval and patient consent

The study protocol was designed in accordance with guidelines outlined in the Declaration of Helsinki and was approved by the ethics committee for human research at Rakuwakai Otowa Hospital, Kyoto, Japan (approval number; 14-003). The methods were performed in accordance with the approved guidelines. Written informed consent was obtained from all patients or their family members.

### Study population

Details regarding clinical data collection, image acquisition, and segmentation and quantification of the ventricles and subarachnoid spaces were described in our prior publications^9^,^10^. In brief, 72 patients diagnosed with NPH based on the enlarged ventricles under normal CSF pressure and the symptoms of gait disturbance, cognitive impairment and urinary disturbance participated in this study. All of them underwent MRI examinations and the CSF tap-test, which consisted of removing ≥30 mL of CSF via a lumbar tap after written informed consent. Eight patients were diagnosed with sNPH after subarachnoid haemorrhage, 7 patients developed sNPH after head injury, and 52 patients were diagnosed with iNPH because a known antecedent cause was absent. Five patients who were diagnosed with congenital aetiology NPH based on the morphological features were excluded from this study. There were relatively few patients with sNPH for statistical analysis, because our NPH centre was consulted for the diagnosis and treatment of a patient once a patient was suspected to have iNPH, but rarely consulted for patients diagnosed with sNPH following subarachnoid haemorrhage. Thirty-one participants ≥60 years old were recruited as controls since they did not have any symptoms of short-stepped gait and/or cognitive impairment, and these controls were confirmed via MRI to be without ventricular dilatation or fluid accumulation, such as subdural haematoma. Controls included patients who were diagnosed with unruptured small intracranial aneurysms or who had a history of small intracerebral haemorrhage or lacunar infarction. Additionally, patients with a history of subarachnoid haemorrhage, head injury, suspected dementia, or neurodegenerative disorders such as Alzheimer’s disease and Parkinson’s disease were excluded from the control group.

### Image acquisition for measurement of segmented CSF volume and parameters

All MRI examinations were performed with a 64-channel 3-Tesla MRI system (MAGNETOM Skyra, Siemens AG, Muenchen, Germany). The sagittal source images of the T2-weighted 3D-SPACE were automatically processed to create 3D volume-rendering reconstruction and multiplanar reconstruction images by using an independent 3D volume analyser workstation (SYNAPSE 3D; FUJIFILM Medical Systems; Tokyo, Japan). The intracranial volumes were segmented by the utilization of the combined techniques of the edge-guided nonlinear interpolation and user-steered live-wire segmentation^58^,^59^. After that, the CSF spaces were automatically segmented from brain parenchyma by using a simple threshold algorithm^60^. The intracranial CSF spaces were manually segmented into the bilateral, third, and fourth ventricles, convexity subarachnoid space, basal cistern and Sylvian fissure, and subarachnoid space in the posterior fossa. Each segmented volume was automatically measured by counting the number of voxels. Brain parenchymal volume was calculated as intracranial volume minus total CSF volume. The volume ratios (%) were calculated as ratios of target volumes to the intracranial volume. The validity, reliability and reproducibility of 3D segmentation and quantification of CSF volumes were assessed and described in the previous article^9^. The Evans Index was measured as the maximal width of the frontal horns of the bilateral ventricles to the maximal width of the internal diameter of the cranium on the basis of the X dimension; an Evans index >0.3 was defined as conventional ventricular dilatation, according to the diagnostic guidelines for iNPH^2^,^3^,^4^,^5^,^6^. Alternatively, the z-Evans index was defined as the maximum z-axial length of the frontal horns of the lateral ventricles to the maximum cranial z-axial length; a z-Evans index >0.4 is considered a useful index for the diagnosis of iNPH^9^. In addition, a callosal angle <90°, measured as the angle of the roof of the bilateral ventricles on the coronal plane, which was perpendicular to the anteroposterior commissure plane on the posterior commissure, was also used as an index specific to iNPH^19^. The fluid attenuated inversion recovery (FLAIR) sequence was also conducted for evaluating the periventricular hyperintensity and deep white matter hyperintensity on the Fazekas rating scale^20^.

### Evidence of direct CSF communication between the lateral ventricles and basal cistern via the choroidal fissure

We subjectively assessed whether there was an opening at the inferior choroidal point of the choroidal fissure using 3D-CISS sequences to evaluate the fine structures in the CSF spaces. Additionally, one patient who was diagnosed with iNPH and who had received a ventriculo-peritoneal shunt four years ago at another hospital needed to undergo shuntography to test the patency of his shunt system because his gait and cognitive function had worsened gradually. A CT scan was performed before and immediately after shuntography, in which a small amount of OMNIPAQUE-240 (iohexol, GE Healthcare) was injected into the left inferior horn of the lateral ventricles via reservoir and ventricular catheter of the shunt system. Furthermore, the actual CSF movement through the thin arachnoid membrane between the inferior horn of the lateral ventricles and the ambient cistern was observed *in situ* during the amygdalohippocampectomy of one patient diagnosed with hippocampal glioma.

### Statistics

Mean values and standard deviations for age, sex, and several MRI parameters were calculated and compared among patients with iNPH, sNPH, and control groups by a Mann-Whitney-Wilcoxon test. The Chi-square test was used to compare the two proportions. Statistical significance was assumed at a probability value (*p*) of less than 0.05. All missing data were treated as deficit data that did not affect other variables. Statistical analysis was performed using R software (version 3.0.1; R Foundation for Statistical Computing, Vienna, Austria; http://www.R-project.org).

## Additional Information

**How to cite this article**: Yamada, S. *et al*. Choroidal fissure acts as an overflow device in cerebrospinal fluid drainage: morphological comparison between idiopathic and secondary normal-pressure hydrocephalus. *Sci. Rep.* **6**, 39070; doi: 10.1038/srep39070 (2016).

**Publisher's note:** Springer Nature remains neutral with regard to jurisdictional claims in published maps and institutional affiliations.

## Supplementary Material

Supplementary Video

Video Legends

## Figures and Tables

**Table 1:** Morphological characteristics of the study population.

	iNPH	sNPH	Control	*P*1	*P*2	*P*3
Total number	52	15	31			
Mean age (years)	76.7 ± 6.9	72.3 ± 10.8	75.8 ± 7.4	0.220	0.546	0.534
Male	31 (60%)	8 (53%)	17 (55%)	0.891	0.844	1.000
Evans index >0.3	41 (79%)	12 (80%)	6 (19%)	1.000	<0.001	<0.001
Z-Evans index >0.4	45 (87%)	7 (47%)	3 (10%)	0.006	<0.001	0.014
Callosal angle <90°	45 (87%)	8 (53%)	5 (16%)	0.005	<0.001	0.023
High convexity tightness	46 (89%)	1 (7%)	1 (3%)	<0.001	<0.001	1.000
Enlarged Sylvian fissure	45 (87%)	2 (13%)	0	<0.001	<0.001	0.191
Opening of the inferior choroidal point	39 (75%)	2 (13%)	0	<0.001	<0.001	0.191
Severe periventricular hyperintensity	44 (85%)	11 (73%)	18 (58%)	0.534	0.015	0.497
Severe deep white matter hyperintensity	36 (69%)	9 (60%)	15 (48%)	0.720	0.098	0.671

The Evans index was measured as the maximal width of the frontal horns of the bilateral ventricles to the maximal width of the internal diameter of the cranium on the basis of the X dimension.

The Z-Evans index was measured as the maximum z-axial length of the frontal horns of the lateral ventricles to the maximum z-axial length of the median skull.

The callosal angle was measured as the angle of the roof of the bilateral ventricles on the coronal plane, which was perpendicular to the anteroposterior commissure plane on the posterior commissure.

Opening of the inferior choroidal point was defined as direct communication of CSF spaces between the inferior horn of the lateral ventricles and the ambient cistern at the inferior choroidal point of the choroidal fissure.

Severe periventricular hyperintensity was defined as large (Grade 2) or extension (Grade 3), or deep white matter hyperintensity according to the grading scales reported by Fazekas *et al*.^20^.

*P*1; probability value of iNPH vs. sNPH.

*P*2; probability value of iNPH vs. control.

*P*3; probability value of sNPH vs. control.

**Table 2:** Mean volumes (mL) and volume ratios (%) of CSF and brain parenchyma.

	iNPH (52)	sNPH (15)	Control (31)	*P*1	*P*2	*P*3
**Total intracranial spaces**	1517 ± 156	1467 ± 147	1487 ± 138	0.310	0.369	0.639
**Brain parenchyma**	1096 ± 119 (72.4%)	1177 ± 134 (80.3%)	1154 ± 147 (77.5%)	0.061	0.017	0.746
**Total CSF**	420.4 ± 84.7 (27.6%)	290.0 ± 85.2 (19.7%)	333.1 ± 94.0 (22.5%)	<0.001	<0.001	0.075
**Total ventricles**	160.8 ± 45.2 (10.6%)	121.4 ± 26.8 (8.2%)	61.3 ± 31.9 (4.2%)	0.002	<0.001	<0.001
Bilateral ventricles	152.9 ± 41.7	112.2 ± 26.7	52.1 ± 26.4	<0.001	<0.001	<0.001
Third ventricle	5.3 ± 1.5	5.2 ± 1.2	3.5 ± 1.3	0.982	<0.001	<0.001
Fourth ventricle	4.1 ± 2.0	4.5 ± 1.1	2.8 ± 1.5	0.181	0.002	<0.001
**Total subarachnoid spaces**	259.6 ± 71.5 (17.1%)	168.4 ± 72.3 (11.5%)	271.7 ± 85.7 (18.3%)	<0.001	0.569	<0.001
Convexity subarachnoid space	76.4 ± 35.4	73.0 ± 35.9	125.8 ± 54.8	0.758	<0.001	<0.001
Basal cistern and Sylvian fissure	120.4 ± 32.7	51.6 ± 35.0	90.7 ± 34.0	<0.001	<0.001	0.001
Posterior fossa	62.8 ± 17.7	43.9 ± 10.2	55.2 ± 19.7	<0.001	0.057	0.038

*P*1; probability value of iNPH vs. sNPH for the Mann-Whitney-Wilcoxon test.

*P*2; probability value of iNPH vs. control for the Mann-Whitney-Wilcoxon test.

*P*3; probability value of sNPH vs. control for the Mann-Whitney-Wilcoxon test.
