# Supplementary material for: Choroidal fissure acts as an overflow device in cerebrospinal fluid drainage: morphological comparison between idiopathic and secondary normal-pressure hydrocephalus
Source: Sci Rep. 2016 Dec 12;6:39070. doi: 10.1038/srep39070 (PMC5150242; doi:10.1038/srep39070)
Supplement: Video Legends [file srep39070-s2.pdf]

Choroidal fissure acts as an overflow device in cerebrospinal fluid drainage: morphological comparison between idiopathic and secondary normal-pressure hydrocephalus

Shigeki Yamada, MD, PhD; Masatsune Ishikawa, MD, PhD; Yasushi Iwamuro, MD, PhD; Kazuo Yamamoto, MD

Video clip:

Microsurgery of a right amygdalohippocampectomy via a transventricular approach

This movie is in mpeg 4 format. The first operative view shows the right hippocampus in situ through the inferior horn of the lateral ventricle. The next high-power field view shows the choroid plexus inside the hippocampal body. The inferior choroidal point of the choroidal fissure was observed after the choroid plexus was displaced outside. When the transparent membrane of the choroidal fissure was extended slightly by the suction tube without microsurgical dissection or opening of the choroidal fissure, we can see to-and-fro CSF movement (yellow arrow) from the ambient cistern to the inferior horn through the transparent membrane of the choroidal fissure.
